# Comparative analysis of complete chloroplast genomes of *Flemingia prostrata* and *Flemingia macrophylla*, two commonly used medicinal plants in southern China

**DOI:** 10.3389/fpls.2025.1591427

**Published:** 2025-08-18

**Authors:** Fan Wei, Yang Lin, Danfeng Tang, Ying Liang, Shuangshuang Qin

**Affiliations:** ^1^ Guangxi Key Laboratory of Medicinal Resources Protection and Genetic Improvement, Guangxi Botanical Garden of Medicinal Plants, Nanning, China; ^2^ National Engineering Research Center for Southwest Endangered Medicinal Materials Resources Development, Guangxi Botanical Garden of Medicinal Plants, Nanning, China

**Keywords:** *Flemingia*, SSR, Fabaceae, phylogenetic analysis, genome comparison

## Abstract

*Flemingia prostrata* and *Flemingia macrophylla*, belonging to the genus *Flemingia*, are ethnomedicinal plants that contain valuable medicinal and nutritional compounds. However, their medicinal materials are frequently confused in the Chinese medicinal materials market. Moreover, molecular genomic resources for this genus remain limited, which hinders phylogenetic studies. In this study, the complete chloroplast (cp) genomes of *F. macrophylla* and *F. prostrata* were sequenced to enable genome comparison and phylogenetic analysis. Both cp genomes exhibited typical quadripartite structures, with genome sizes of 152,937 bp for *F. macrophylla* and 153,033 bp for *F. prostrata*. Each genome consisted of a large single copy (LSC) region (83,594 and 83,701 bp, respectively), a small single copy (SSC) region (17,773 and 17,776 bp, respectively), and two inverted repeats (IR) regions (50,570 and 51,556 bp, respectively). A total of 129 genes were annotated in each cp genome, including 8 ribosomal RNAs, 83 protein-coding genes, and 37 transfer RNAs. Comparative analysis revealed that although the overall genome structure, codon usage bias, simple sequence repeats (SSRs), and dispersed repetitive sequences were relatively conserved between the two cp genomes, certain genomic variations were present. Specifically, 286 SNPs and 104 indels were identified, and *psaJ-rps18* showed the highest variability and could serve as potential DNA barcode regions. Furthermore, phylogenetic analysis supported a close evolutionary relationship between the genus *Flemingia* and *Cajanus*. Divergence time estimation suggested that *F. macrophylla* and *F. prostrata* diverged approximately 0.26 million years ago (Mya). Finally, we successfully distinguished the two species using SSR markers. This study lays the foundation for enriching the molecular data and phylogenetic insights of this genus, as well as for the safe application of its medicinal materials.

## 1 Introduction

*Flemingia* Roxb. ex W. T. Ait. belongs to Papilionoideae, a subfamily of the Fabaceae. There are approximately 40 species of *Flemingia* widely distributed in the tropics of Asia, Africa, and Oceania of the world. China has 16 species and 1 variety, mainly distributed in Southwest, Southeast, and Central South China (Flora Reipublicae Popularis Sinicae, http://www.iplant.cn/frps). Most plants of *Flemingia* have medicinal values, and 6 species of *Flemingia* are native to China, which has a long history of use in folk medicine (Jia and Li, 2005). For example, *Flemingia prostrata* (*F. prostrata*) and *Flemingia involucrata* (*F. involucrata*) have the original recordings in “Zhiwu Mingshi Tukao”, written by Wu Qi-jun in the Qing Dynasty of China (Wu, 2003, 1991). *F. prostrata* is documented in “Annals of Chinese Ethnic Medicine” (China Institute for the Control of Pharmaceutical and Biological Products and Yunnan Institute for Drug Control, 1990), and *Flemingia macrophylla* (*F. macrophylla*) is included in “Annals of Zhuang Nationality Medicine in China” (Zhu and Wang, 2003).

To date, a total of 197 compounds have been successfully separated and identified from *Flemingia*, including flavonoids, saponins, anthraquinones, triterpenes, volatile foril, etc. Furthermore, contemporary pharmacological research has revealed numerous pharmacological properties associated with the extracts and individual compounds (primarily flavonoids). These properties include analgesic and anti-inflammatory effects, antibacterial and antifungal activities, estrogenic effects, skin protection, neuroprotection, and hepatoprotective effects (Ho et al., 2011; Yan et al., 2016).

**Figure 1:**
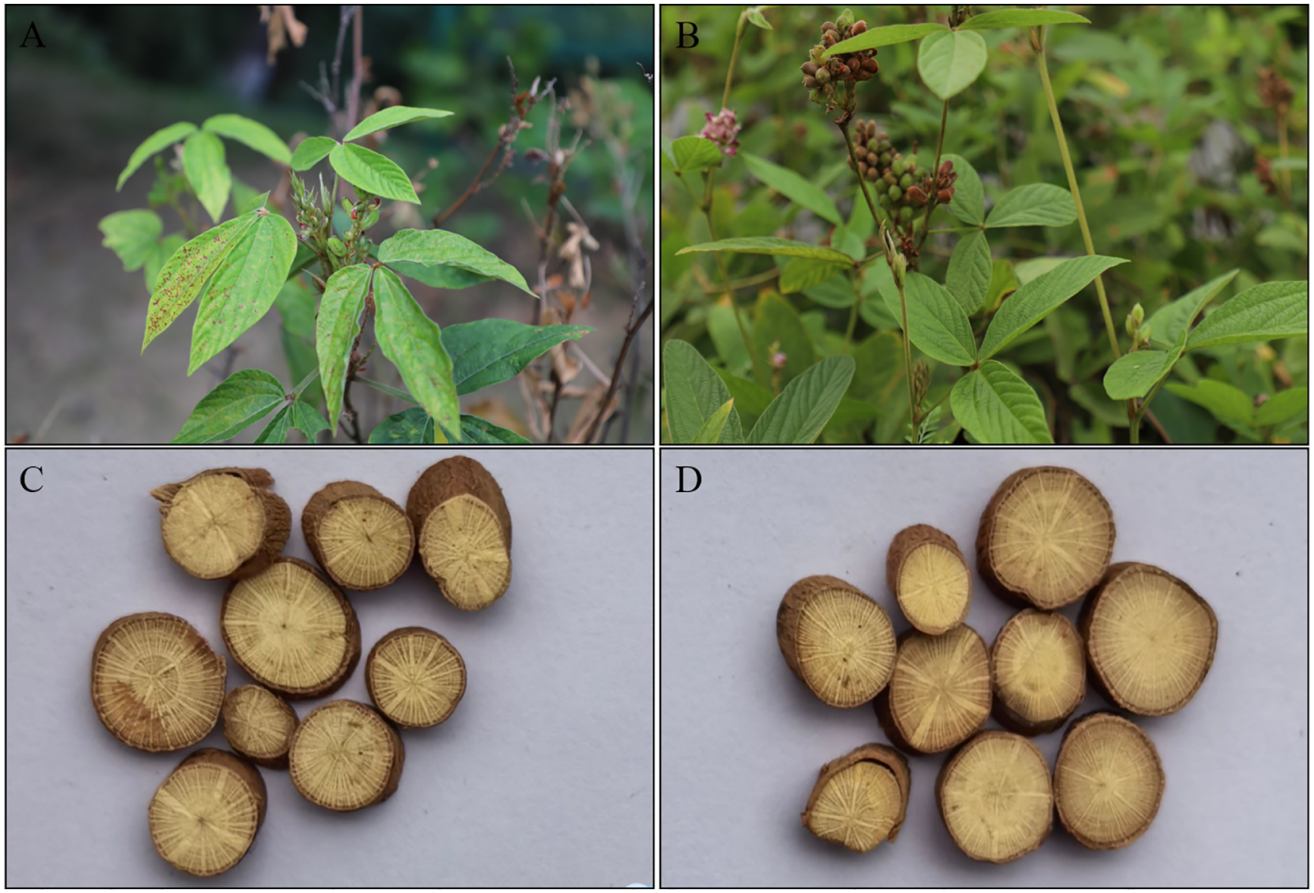
Plant morphology of *F. macrophylla* and *F. prostrata.* **(A, C)** The plant morphology and root cross section of *F. macrophylla*. **(B, D)** The plant morphology and root cross section of *F. prostrata*.

Currently, certain *Flemingia* species are sold as mixtures in herbal markets for use in Traditional Chinese Medicine (TCM), adversely affecting their efficacy and safety. Moreover, there are currently no national quality standards for *Flemingia* medical plants, with only *F. prostrata*, *F. macrophylla*, and *Flemingia ferruginea* (*F. ferruginea*) listed in the appendix of “The 2020 edition of the Pharmacopoeia of the People’s Republic of China (China Pharmacopoeia Committee, 2020). Of these, *F. prostrata* and *F. macrophylla* are widely distributed in southwest of China, such as Yunnan, Guangxi, Guangdong, Jiangxi, Fujian, Taiwan, Hubei, Hunan, and Sichuan provinces, with Guangxi and Yunnan being particularly notable. The dried roots of these plants exhibit efficacy in alleviating rheumatism, arthritis, and gynecological pain, and possess hepatoprotective, estrogenic, neuroprotective, cytotoxic, and antitumor activities (Lai et al., 2013; Niu et al., 2021). They are also widely used by Hani, Dai, Zhuang, and Yao ethnic minorities in China (Song et al., 2001). Although both *F. prostrata* and *F. macrophylla* are listed in the Chinese Pharmacopoeia, they differ in practical applications. For instance, *F. macrophylla* is primarily used in Qianjin Pharmaceutical’s Fuke Qianjin Pian (Gynecological Qianjin Tablet), while *F. prostrata* is mainly utilized in ethnic medicines in Hunan Province (Zheng et al., 2022; She et al., 2022). Moreover, the use of *F. prostrata* and *F. macrophylla* also differs in various ethnic medicines in China. For example, *F. prostrata* is primarily used in Yao medicine and Dong medicine, while *F. macrophylla* is mainly utilized in Zhuang medicine and Dai medicine. Nevertheless, due to their similar characteristics, especially the roots, the medicinal materials of *F. prostrata* and *F. macrophylla* are often confused in the Chinese medicinal materials market (**Figure 1**). Therefore, ensuring the authenticity of these herbs is crucial for their effective use. However, it is challenging to visually differentiate *F. prostrata* from *F. macrophylla* based on macroscopic characteristics. Hence, there is an urgent need for robust and advanced DNA technology to differentiate *F. prostrata* from *F. macrophylla*.

DNA barcoding is a molecular technique for species identification that utilizes a standardized, short segment of DNA sequence (Kress et al., 2005; Schindel and Miller, 2005). The chloroplast (cp) genome is shorter and easier to amplify than traditional DNA barcodes, and it contains more information on variable sites. This makes it a powerful molecular marker capable of deeply resolving subtle but crucial genetic differences among different species. Chloroplast non-coding regions have been established as DNA barcodes in plants for conservation and mutation studies (Dong et al., 2012, 2014). Thus, genomic information from the chloroplasts of *F. prostrata* and *F. macrophylla* can serve as candidate DNA barcodes for distinguishing between these species. Additionally, to date, there have been few reports on the cp genome information of *F. prostrata* and *F. macrophylla*, especially regarding the comparative analysis of their cp genomes.

In this study, we employed high-throughput sequencing technology to sequence the cp genomes of *F. prostrata* and *F. macrophylla*, and conducted characterization and comparative analysis of their cp genomes. The aim was to conduct species identification and differentiation of *F. prostrata* and *F. macrophylla* at the molecular level, thereby enhancing the safety of their medicinal use.

## 2 Materials and methods

### 2.1 DNA extraction and sequencing

The *F. prostrata* and *F. macrophylla* plants were identified by associate researcher Ying Hu and the voucher specimens were preserved in Guangxi Botanical Garden of Medicinal Plants. Fresh leaves of *F. macrophylla* and *F. prostrata* were obtained from the Guangxi Botanical Garden of Medicinal Plants (Nanning, China). The genomic DNA extraction Kit (TIANGEN, Beijing, China) was employed for extracting the complete genomes of two *Flemingia* species. The sequencing library was constructed after genomic DNA quality assessment and PCR amplification. After qualifying the library, we performed sequencing using the Illumina Novaseq 6000 platform, and the read length for pairwise sequencing (PE) was 150 bp.

### 2.2 Chloroplast genome assembly and annotation analyses

First, Fastp (v 0.20.0) (https://github.com/OpenGene/fastp) was utilized to obtain clean reads by removing the joint sequences, adapters, and low-quality reads from the raw data. After quality control, the core module of the cp genomes for two *Flemingia* species was assembled using the SPAdes-3.13.0 (http://cab.spbu.ru/software/spades/) software with kmers of 55, 87, and 122. The assembly process was not dependent on a reference genome, highlighting its independent and original nature.

After *de novo* assembly, we carried out three methods to improve the annotation accuracy. First, we compared the reads to the genomes, calculating the genome coverage, inserted fragment size, and other information. Then, the target genomes were compared with the reference sequence genomes and analyzed for genome conservation and rearrangement. Finally, we compared the differences in structure between target genomes and the reference sequences.

To enhance the annotation efficiency, two approaches were employed for annotating the cp genomes. Initially, Prodigal v2.6.3 (https://www.github.com/hyattpd/Prodigal) was utilized for annotating the coding sequences within the cp genomes. Further, the ribosomal RNAs and transfer RNAs were predicted using Hmmer v3.1b2 (http://www.hmmer.org/) and Aragorn v1.2.38 (http://130.235.244.92/ARAGORN/) software, respectively. Subsequently, the gene sequences were obtained from the NCBI database using homolog sequences from similar species as a reference. Afterward, the assembled sequence was compared to the gene sequences using Blast v2.6 (https://blast.ncbi.nlm.nih.gov/Blast.cgi). Finally, the genes identified in both sets of annotations were manually reviewed, with any inaccurate or duplicate entries removed, the multiexon boundary established and the annotation conducted. The circular cp genome maps were annotated using the online tool OGDRAW (https://chlorobox.mpimp-golm.mpg.de/OGDraw.html).

### 2.3 Scattered repeat sequence analysis

Four repeat sequence types in two *Flemingia* species, including forward, palindromic, reverse, and complement, were found utilizing Vmatch v2.3.0 (http://www.vmatch.de/) combined Perl script and set a minimum repeat size of 30 bp and hamming distance of 3. The analysis of SSRs was conducted on the cp genomes of two *Flemingia* species using MISA v1.0 (MIcroSAtellite identification chloroplasttool, http://pgrc.ipk-gatersleben.de/misa/misa.html) with the parameter that the minimum number of repeats required for identification being 8, 5, 3, 3, 3, and 3 for mono-, di-, tri-, tetra-, penta-, and hexanucleotides, respectively.

### 2.4 Codon usage analysis

For the identification of codon usage patterns, all coding sequences (CDSs) were analyzed to calculate the relative synonymous codon usage (RSCU) and the effective number of codons (ENC) using CodonW (Peden, 1999). GC3s refers to the G+C content at the third position of synonymous codons in CDS sequences, normalized by the total number of such bases excluding Met and Trp codons, and was computed using the CUSP program in EMBOSS Explorer (https://www.bioinformatics.nl/embossexplorer/). An ENC-plot was plotted with GC3s as the horizontal coordinate and ENC as the vertical coordinate to elucidate the factors influencing codon usage bias within a gene or genome.

### 2.5 Genome comparative analysis

For comparative analysis, the chloroplast genome sequences of three other *Flemingia* species: *Flemingia stricta* (NC086853.1), *Flemingia* sp. (OR733289) and *Flemingia philippinensi*s (PV464028.1) were obtained from the NCBI database. To assess the genome divergence among the five *Flemingia* cp genomes (*F. macrophylla*, *F. prostrata, Flemingia stricta*, *Flemingia* sp. and *Flemingia philippinensi*s), the MAUVE (Darling et al., 2004) program was employed using *F. macrophylla* as the reference. Additionally, IRscope (https://irscope.shinyapps.io/irapp/) was utilized to examine variations in the boundary regions between the LSC, IRB, SSC, and IRA regions across the five *Flemigia* species.

### 2.6 Nucleotide diversity and gene selective pressure analyses

The nucleotide diversity (Pi) of the five *Flemingia* species cp genomes was carried out utilizing the DnaSP (v6) (Rozas et al., 2017), on a 200 bp window length and 50 bp step size. The KaKs_Calculator v2.0 (Zhang et al., 2006) was used to calculate the rates of synonymous (Ks) and nonsynonymous (Ka) substitutions to determine the selective pressure for the shared protein-coding genes.

### 2.7 Phylogenetic relationship analysis and divergence time estimation

The cp genomes of thirty-four species, representing six subfamilies of Fabaceae (*Caesalpinioideae, Papilionoideae, Cercidoideae, Duparquetioideae, Detarioideae*, and *Dialioideae*), including those of *F. stricta*, *Flemingia* sp. and *F. philippinensi*s, as well as two outgroup cp genomes from *Cannabis* and *Morus*, were obtained from the NCBI database for analysis (**Supplementary Table S1**). To directly compare the phylogenetic resolution offered by a broad chloroplast dataset versus a four-gene barcode set, we first extracted and concatenated the coding sequences of 57 chloroplast genes into a single alignment and, in parallel, concatenated the *rbcL*, *matK*, *trnL*-*trnF* and *ndhF* regions. Both datasets were aligned using MAFFT v7.310, after which we inferred maximum-likelihood (ML) phylogenies in RAxML v8.2.12 under the GTR+GAMMA model with 1,000 rapid bootstrap replicates (Katoh and Standley, 2013, Alexandros Stamatakis, 2014). Finally, we employed the tanglegram function in the R package phytools (Revell, 2012) to visually juxtapose and evaluate the topological congruence between the two trees.

The ML tree constructed from the CDS of 57 chloroplast genes of Fabaceae was used as a starting tree for MCMC run. MCMC run was set 1,000,000 generations, sampling every 100 generations, and removing the first 10% generations as burn in. Divergence time estimation was calculated by parameters of clock = 2 and model = 0, with 95% highest posterior density (HPD) intervals.

### 2.8 cpSSR molecular identification

By comparing the cp genome sequences of *F. prostrata* and *F. macrophylla*, 75 differential cpSSR loci were identified. Ten of these loci were randomly selected for cpSSR primer design, and the corresponding primers are listed in **Supplementary Table S2**. DNA was extracted from five individual plants of each specie (*F. prostrata* and *F. macrophylla*), and PCR amplification was performed using the following reaction system: 2×Taq PCR MasterMix 10 µL, forward primer 2 µL (10 µmol/L), reverse primer 2 µL (10 µmol/L), DNA 1 µL, and ddH_2_O 5 µL. The PCR amplification procedure consisted of an initial denaturation at 95°C for 2 min, followed by 26 cycles of denaturation at 94°C for 40 s, annealing at 56°C for 45 s, and extension at 72°C for 1 min. A final extension step was carried out at 72°C for 7 min. The PCR products were subjected to electrophoresis on an 8% non-denaturing polyacrylamide gel at 120 V, 400 mA for 30 min and then 180 V, 400 mA for 1 h 15 min, followed by staining with silver nitrate.

## 3 Results

### 3.1 Complete cp genome features of *F. macrophylla* and *F. prostrata*

**Figure 2:**
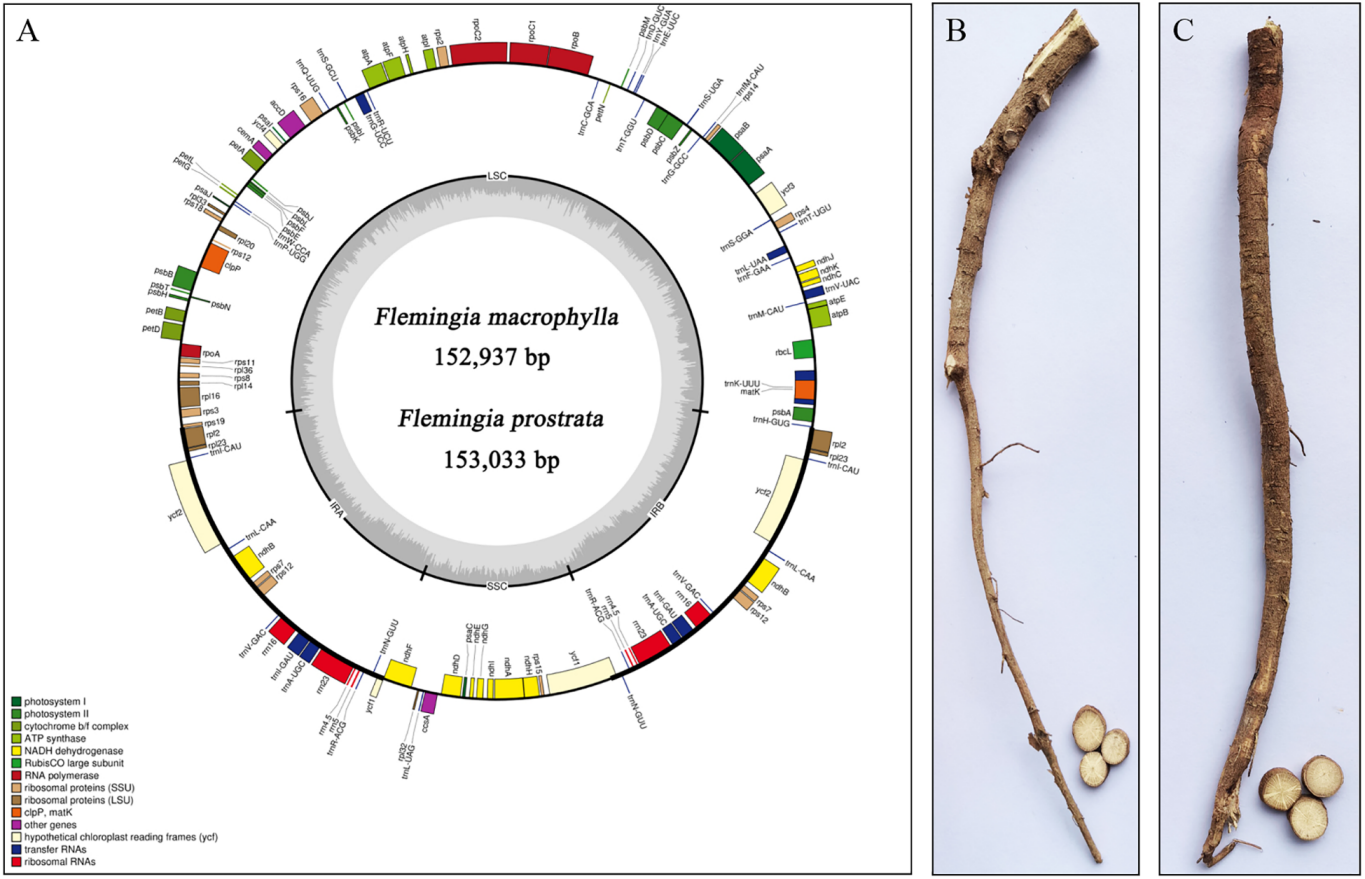
**(A)** Maps of the chloroplast genome of *F*. *macrophylla* and *F. prostrata*. The genes in the clockwise direction fill the inner circle, and the outer circle contains genes in the counterclockwise direction. Different colours represent different genes in different functional groups. The lighter grey shows the A + T content, and the darker grey in the inner circle indicates the G + C content. **(B)** The root of *F*. *macrophylla.* **(C)** The root of *F*. *prostrata*.

The cp genome sequences of *F. macrophylla* and *F. prostrata* have been deposited in the National Center for Biotechnology Information (*Flemingia macrophylla*, NC_065865.1; *Flemingia prostrata*, NC_065863.1). The cp genomes of *F. macrophylla* and *F. prostrata* were 152,937 bp and 153,033 bp in length, respectively. Both genomes exhibited a complete circular structure composed of four-segment structure: a pair of inverted repeats (IRs) that segregated the genome into large single copy (LSC) and small single copy (SSC) regions (**Figure 2**). The LSC regions were 83,594 bp and 83,701 bp in length, while the SSC regions were 17,773 bp and 17,776 bp in length, respectively. The IRs were 50,570 bp and 51,556 bp in length. The two *Flemingia* species exhibited nearly identical GC contents across their LSC, SSC, and IR regions (**Table 1**). However, within each species, the IR region exhibited a higher GC content t compared to the LSC and SSC regions. The cp genomes of both *Flemingia* species contained a total of 129 genes, including 8 ribosomal RNA genes, 83 protein-coding genes, 37 transfer RNAs, and 1 pseudogene (**Table 2**). Among these genes, 19 contained one intron, 4 contained two introns, and 18 had two copies.

**Table 1:** Summary statistics for the assembly of two *Flemingia* species chloroplast genomes.

Genome features	*F. macrophylla*	*F. prostrata*
Genome size (bp)	152937	153033
LSC (bp)	83594	83701
SSC (bp)	17773	17776
IRa (bp)	25785	25778
IRb (bp)	25785	25778
Number of genes	129 (110)	129 (110)
Protein genes (unique)	83 (76)	83 (76)
tRNA genes (unique)	37 (30)	37 (30)
rRNA genes (unique)	8 (4)	8 (4)
GC content (%)	35.12	35.10
GC content in LSC (%)	32.45	32.41
GC content in SSC (%)	28.39	28.38
GC content in IRa (%)	41.78	41.78
GC content in IRb (%)	41.78	41.78

**Table 2:** List of annotated genes in *F. macrophylla* and *F. prostrata* chloroplast genomes.

Category	Gene group	Gene name
Photosynthesis	Subunits of photosystem I	*psaA,psaB,psaC,psaI,psaJ*
	Subunits of photosystem II	*psbA,psbB,psbC,psbD,psbE,psbF,psbH,psbI,psbJ,psbK,psbL,psbM,psbN,psbT,psbZ*
	Subunits of NADH dehydrogenase	ndhA*,ndhB*(2),ndhC,ndhD,ndhE,ndhF,ndhG,ndhH,ndhI,ndhJ,ndhK
	Subunits of cytochrome b/f complex	petA,petB*,petD*,petG,petL,petN
	Subunits of ATP synthase	atpA,atpB,atpE,atpF*,atpH,atpI
	Large subunit of rubisco	*rbcL*
Self-replication	Proteins of large ribosomal subunit	#rpl33,rpl14,rpl16*,rpl2*(2),rpl20,rpl23(2),rpl32,rpl36
	Proteins of small ribosomal subunit	rps11,rps12**(2),rps14,rps15,rps16*,rps18,rps19,rps2,rps3,rps4,rps7(2),rps8
	Subunits of RNA polymerase	rpoA,rpoB,rpoC1*,rpoC2
	Ribosomal RNAs	*rrn16(2),rrn23(2),rrn4.5(2),rrn5(2)*
	Transfer RNAs	trnA-UGC*(2),trnC-GCA,trnD-GUC,trnE-UUC,trnF-GAA,trnG-GCC,trnG-UCC*,trnH-GUG,trnI-CAU(2),trnI-GAU*(2),trnK-UUU*,trnL-CAA(2),trnL-UAA*,trnL-UAG,trnM-CAU,trnN-GUU(2),trnP-UGG,trnQ-UUG,trnR-ACG(2),trnR-UCU,trnS-GCU,trnS-GGA,trnS-UGA,trnT-GGU,trnT-UGU,trnV-GAC(2),trnV-UAC*,trnW-CCA,trnY-GUA,trnfM-CAU
Other genes	Maturase	*matK*
	Protease	clpP**
	Envelope membrane protein	*cemA*
	Acetyl-CoA carboxylase	*accD*
	c-type cytochrome synthesis gene	*ccsA*
Genes of unknown function	Conserved hypothetical chloroplast ORF	ycf1(2),ycf2(2),ycf3**,ycf4

Gene*: Gene with one introns; Gene**: Gene with two introns; #Gene: Pseudo gene; Gene(2): Number of copies of multi-copy genes.

### 3.2 Repeated sequence analysis

**Figure 3:**
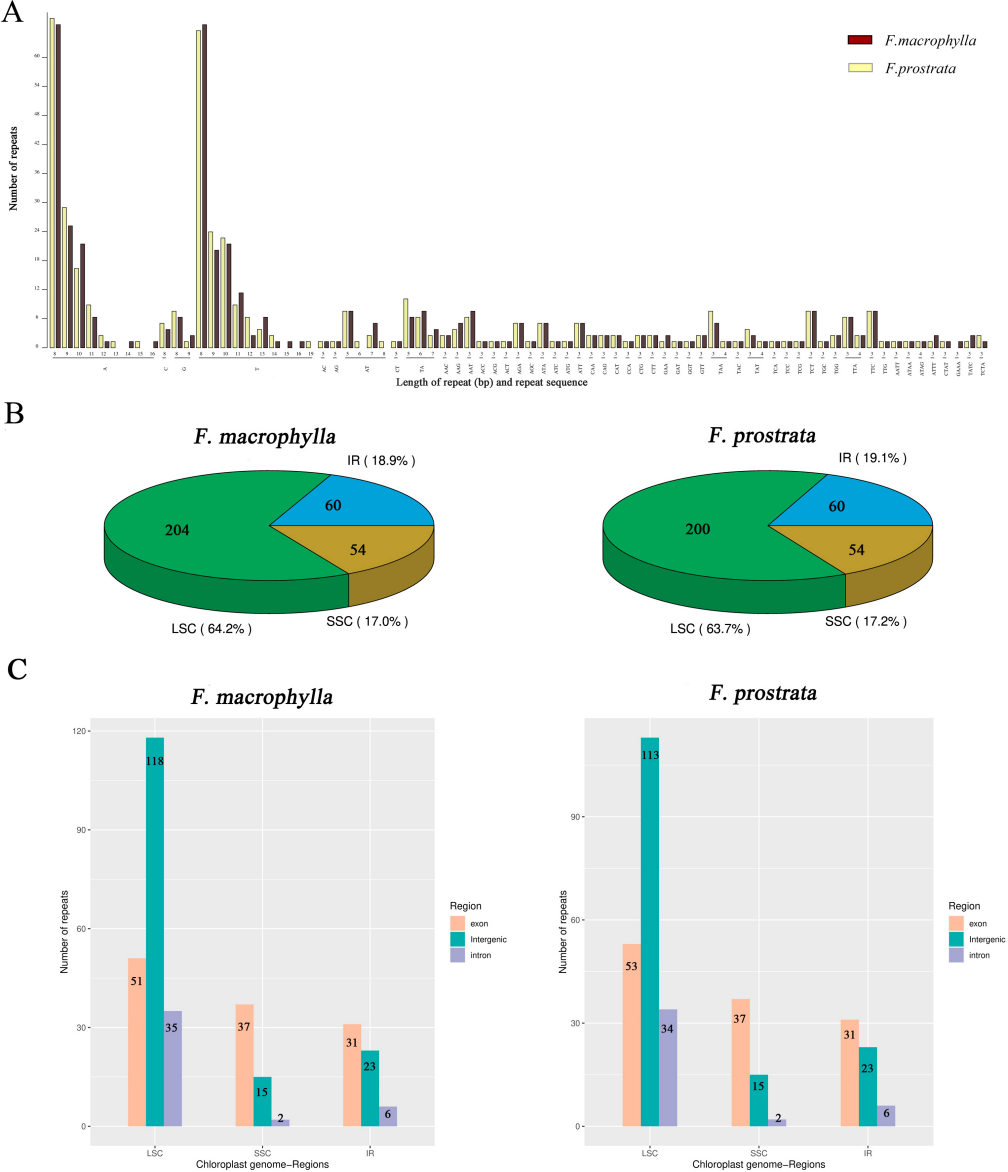
Analysis of SSRs in the chloroplast genomes of *F*. *macrophylla* and *F*. *prostrata*. **(A)** Distribution statistics of SSRs based on type and length. **(B)** Proportional distribution of SSRs in the LSC, SSC, and IR regions. **(C)** Frequency of SSRs in exon, intergenic, and intron regions across the LSC, SSC, and IR regions.

**Figure 4:**
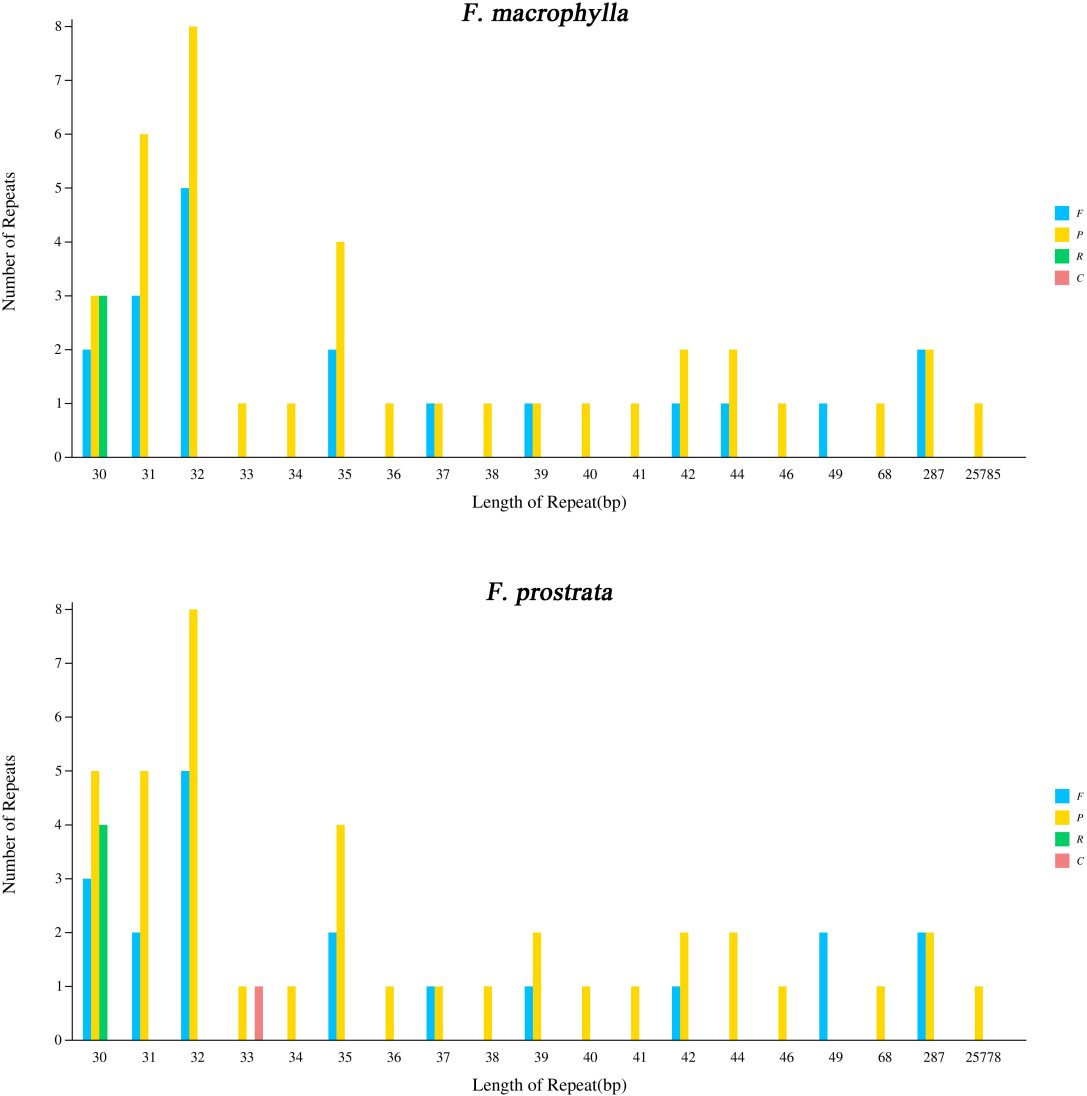
Analysis of scattered repeat sequences in the chloroplast genome of *F. macrophylla* and *F. prostrata.* F, forward; P, palindromic; R, reverse; C, complement.

In this study, four types of simple sequence repeats (SSRs) (mono-, di-, tri, and tetra) were identified in the chloroplast genomes of two medicinal *Flemingia* species. A total of 337 SSRs (219 mono-, 28 di-, 82 tri, and 8 tetra) were found in the cp genome of *F. macrophylla*, while 332 SSRs (213 mono-, 27 di-, 82 tri, and 10 tetras) were identified in *F. prostrata* (**Figure 3A**; **Supplementary Table S3**). Compared to *F. prostrata*, *F. macrophylla* contained six additional mono-SSRs, one more di-SSRs, and two fewer tri-SSRs. Furthermore, SSRs were predominantly distributed in the LSC region, accounting for 63.7% and 63.2% of the total SSRs in *F. macrophylla* and *F. prostrata*, respectively. In contrast, 17.0% and 17.2% were in the SSC region, and 18.9% and 19.1% in the IR region for the two species, respectively (**Figure 3B**). Specifically, within the LSC region, 51, 35, and 118 SSRs were found in the exon, intron, and intergenic regions of *F. macrophylla*, compared to 53, 34, and 113 in *F. prostrata*. Both species exhibited 37, 2, and 15 SSRs in the exon, intron, and intergenic regions of the LSC, respectively, and 31, 6, and 23 SSRs in the corresponding regions of the IR (**Figure 3C**). Additionally, a total of 60 and 64 scattered repeat sequences (including forward, palindromic, reverse, and complement) were identified in the cp genomes of *F. macrophylla* and *F. prostrata*, respectively. Notably, *F. macrophylla* lacked complementary repeats, while *F. prostrata* contained one. Specifically, *F. macrophylla* harbored 19 forward, 38 palindromic, and 3 reverse repeats, whereas *F. prostrata* had the same number of forward repeats, two more palindromic repeats, and one fewer reverse repeat (**Figure 4**).

### 3.3 Analysis of codon preference

**Figure 5:**
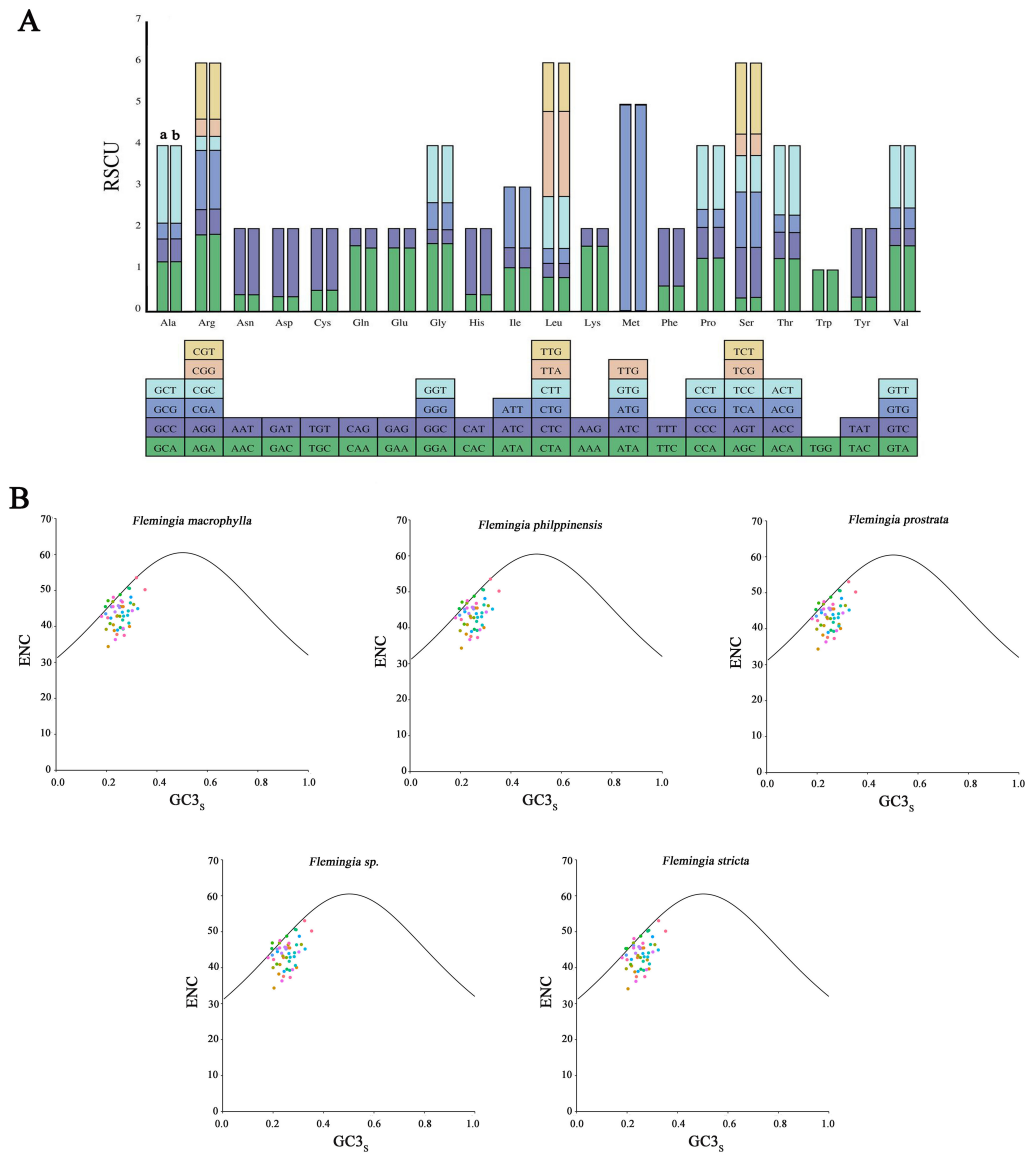
Analysis of codon usage. **(A)** Relative synonymous codon usage (RSCU) of 20 amino acid codons in all protein-coding genes of the chloroplast genome of *F*. *macrophylla* and *F. prostrata.* a: *F*. *macrophylla*, b: *F*. *prostrata.* **(B)** Analysis of ENC-plot of *F*. *macrophylla*, *F*. *philippinensis*, *F*. *prostrata*, *F*. *stricta*, and *Flemingia* sp.

At the molecular level, within the genetic code, multiple codons can code for the same amino acid, these are known as synonymous codons. The relative synonymous codon usage (RSCU) value serves as a quantitative indicator of the preference or bias for specific synonymous codons within a gene or genome. In this study, RSCU values were calculated based on the protein-coding sequences of the cp genomes of *F. macrophylla* and *F. prostrata*. The protein sequences of these two species comprised 26,146 and 26,151 codons, respectively, including termination codon. As shown in **Figure 5A** and **Supplementary Table S4**, both *Flemingia* species utilized 64 codon types to encode 21 amino acid types. The codon counts ranged from 1 to 1,195 in *F. macrophylla* and from 1 to 1,190 in *F. prostrata*. The amino acid tryptophan (Trp) was encoded by only one codon, whereas the remaining amino acids were encoded by 2–6 codons. In both *F. macrophylla* and *F. prostrata*, leucine exhibited the highest codon usage (10.522% and 10.523%, respectively), while cysteine showed the lowest (1.132% and 1.120% of the total number of codons, respectively). Moreover, codon usage was nearly identical between the cp genomes of the two species, with RSCU values ranging from 0.0085 to 4.9655. Several amino acids exhibited codon bias, except for tryptophan (UGG), which had an RSCU value of exactly 1, indicating no codon preference. Overall, these results suggest a high degree of conservation in the cp genomes of *F. macrophylla* and *F. prostrata*.

To further investigate the factors influencing codon usage bias, ENc and GC3s plots were employed. As illustrated in **Figure 5B**, the ENc-GC3 patterns in the two genomes and those of three other *Flemingia* species exhibit notable similarities. The ENc values ranged from 34.14 to 53.52, reflecting diverse trends in codon preference. As indicated by the average ENc values (43.61-43.76) in **Supplementary Table S5**, most coding sequences displayed only weak codon bias. While a limited number of genes were located above or near the expected curve, the majority fell within the lower region of the curve. This suggests that, although mutation pressure influences codon usage patterns, other factors, particularly natural selection, also play a significant role.

### 3.4 Comparative analysis of five complete chloroplast genomes within *Flemingia*

**Figure 6:**
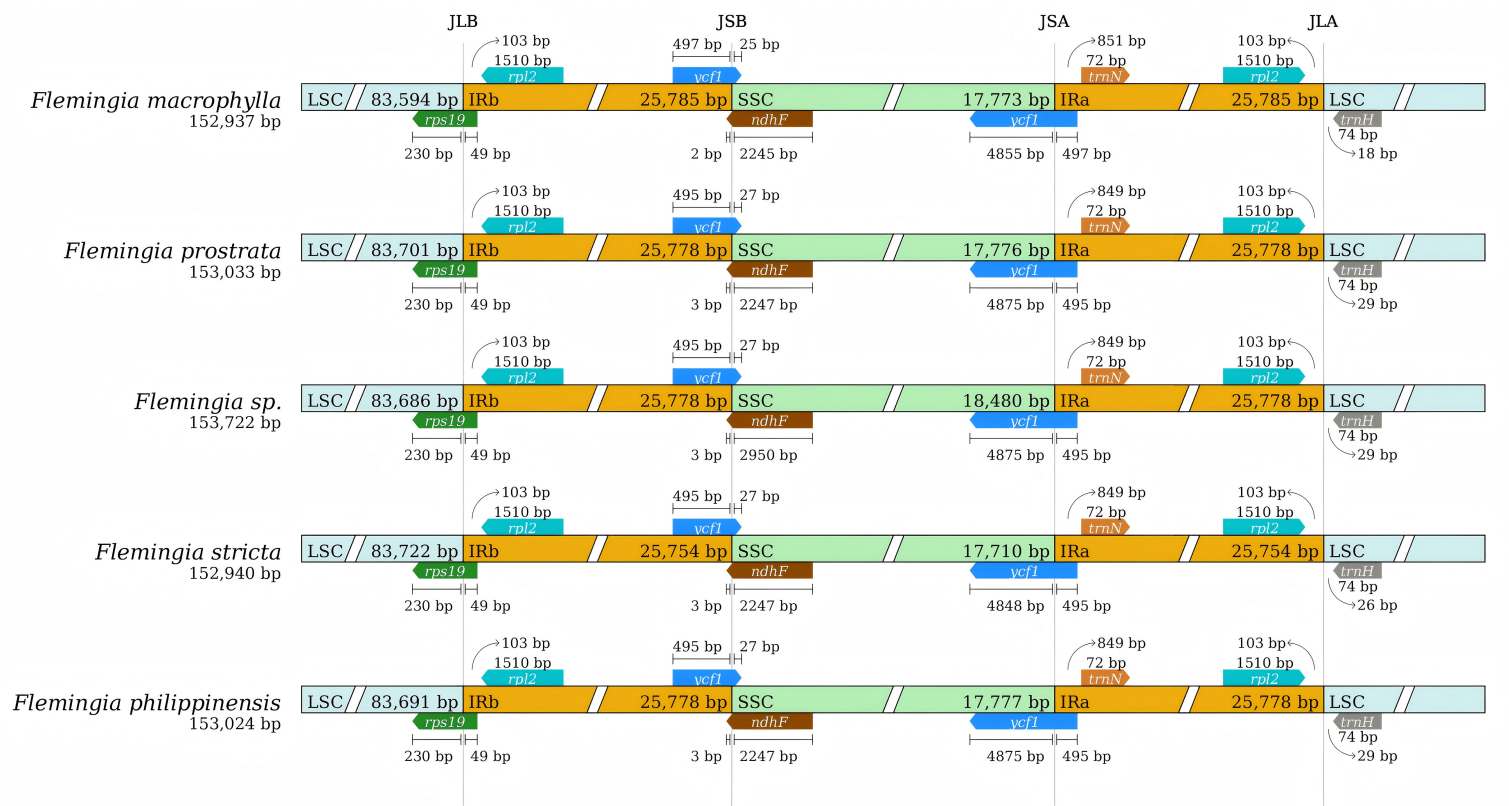
Comparison of the large single copy (LSC), small single copy (SSC), and inverted repeat (IR) regions in chloroplast genomes of *F. macrophylla, F. prostrata*, *F. stricta*, *Flemingia* sp. and *F. philippinensis.* Genes are denoted by colored boxes. The gaps between the genes and the boundaries are indicated by the base lengths (bp).

The contraction and expansion of the IR region boundaries are major factors contributing to variances in cp genome size, illustrating critical roles in evolution processes (Wang et al., 2008). A comparison was conducted between the IR/LSC and IR/SSC junction regions of *F. macrophylla*, *F. prostrata, F. stricta*, *Flemingia* sp. and *F. philippinensis* to identify potential instances of IR boundary expansion or contraction (**Figure 6**). Among the five cp genomes, the genes *rps19*, *ycf1*/*ndhF*, and *ycf1* were found at the LSC/IRb (JLB), IRb/SSC (JSB), and SSC/IRa (JSA) junctions, respectively. The *rpl2* gene is entirely located within the IR regions, positioned 103 bp from JLB junction. The *rps19* gene is primarily located in the LSC region and spans 49 bp into the JLB junction. Furthermore, in the cp genomes of *F. prostrata*, *Flemingia* sp., and *F. philippinensis*, the distance between *trnH* and the JLA junction is 29 bp, whereas it is 18 bp in *F. macrophylla* and 26 bp in *F. stricta*. In *F. macrophylla*, *trnN* is located 851 bp from the JSA junction, while in the other four species, this distance remains consistent at 849 bp. The *ndhF* gene is predominantly located in the SSC region but extends into the JSB junction by 2 bp in *F. macrophylla* and by 3 bp in the remaining four *Flemingia* species. The *ycf1* gene spans both the JSA and JSB junctions across all analyzed genomes. Within the IRb region, the *ycf1* gene spans the JSB junction by 25 bp in *F. macrophylla* and by 27 bp in the other four species. Within the IRa, region, the *ycf1* gene spans the JSA junction by 4,875 bp in *F. prostrata*, *Flemingia* sp., and *F. philippinensis*, and by 4,855 and 4,848 in *F. macrophylla* and *F. macrophylla*, respectively. In conclusion, the IR/LSC borders of these five chloroplast genomes of *Flemingia* were relatively conserved and similar, but the IR/SSC borders exhibited variations.

**Figure 7:**
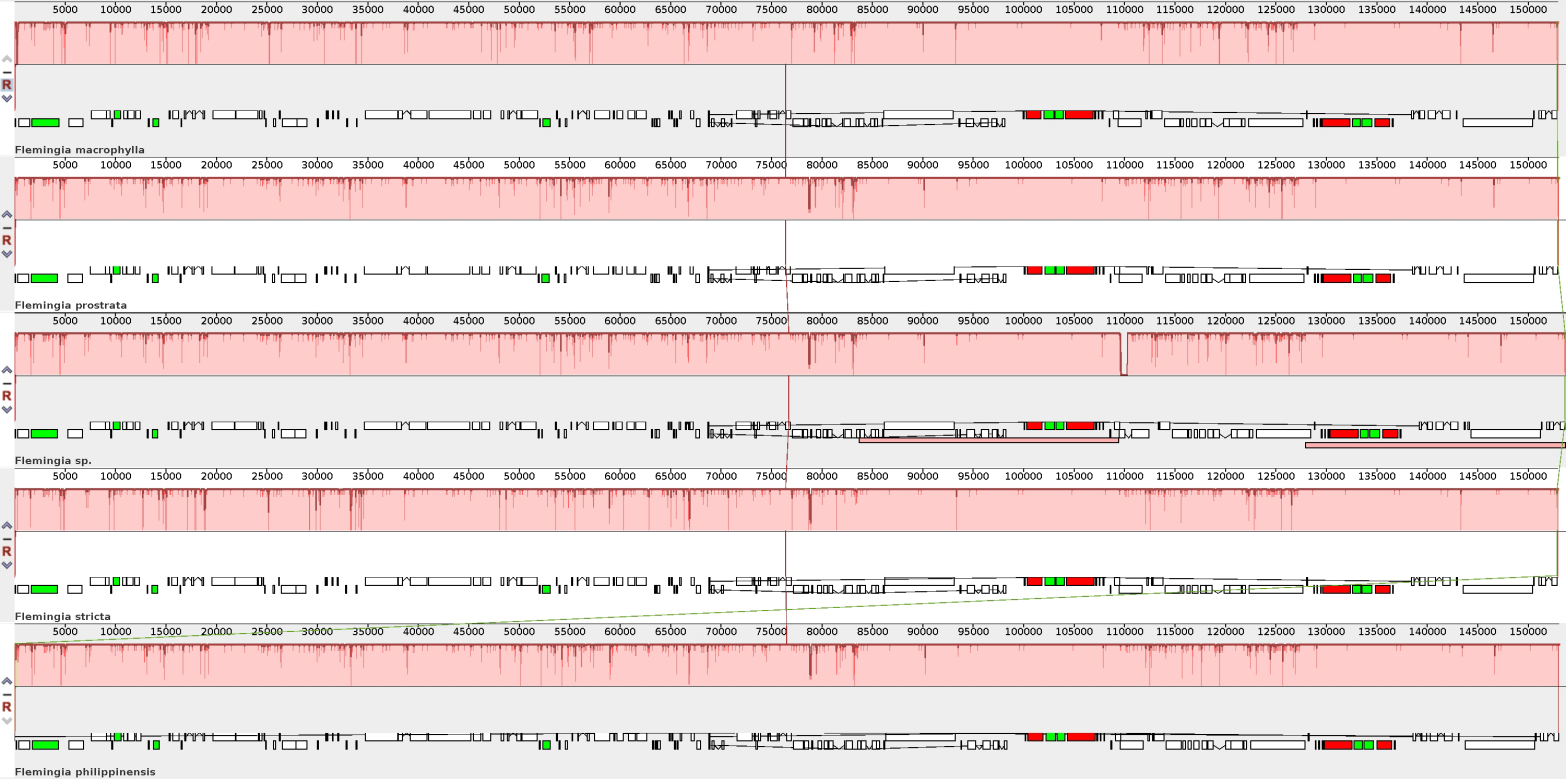
Comparison of the chloroplast genome structures among *F. macrophylla*, *F. prostrata*, *F. stricta*, *Flemingia* sp. and *F. philippinensis* based on the Mauve alignment analysis. The chloroplast genome of *F. macrophylla* is used as the reference and displayed at the top. Consistently colored bars connected with lines in the figure indicate locally collinear blocks (LCBs), which represent clusters of homologous genes. The vertical lines of color of the bar structure indicate the relative conservation of the position, i.e. similarity. The small boxes below each chloroplast genome indicate genes; upper and lower boxes are transcribed counterclockwise and clockwise, respectively. White green box indicate CDS, red boxes indicate iRNA, green box indicate tRNA.

To verify the possibility of genome divergence, sequence identity was calculated for the five *Flemingia* cp genomes using the program MAUVE with *F. macrophylla* as a reference (**Figure 7**). The results indicated that the five cp genomes exhibited a high degree of consistency and conservation of gene order, although there were also certain levels of sequence variation.

### 3.5 Highly divergence regions and selective pressure analysis of *Flemingia*

**Figure 8:**
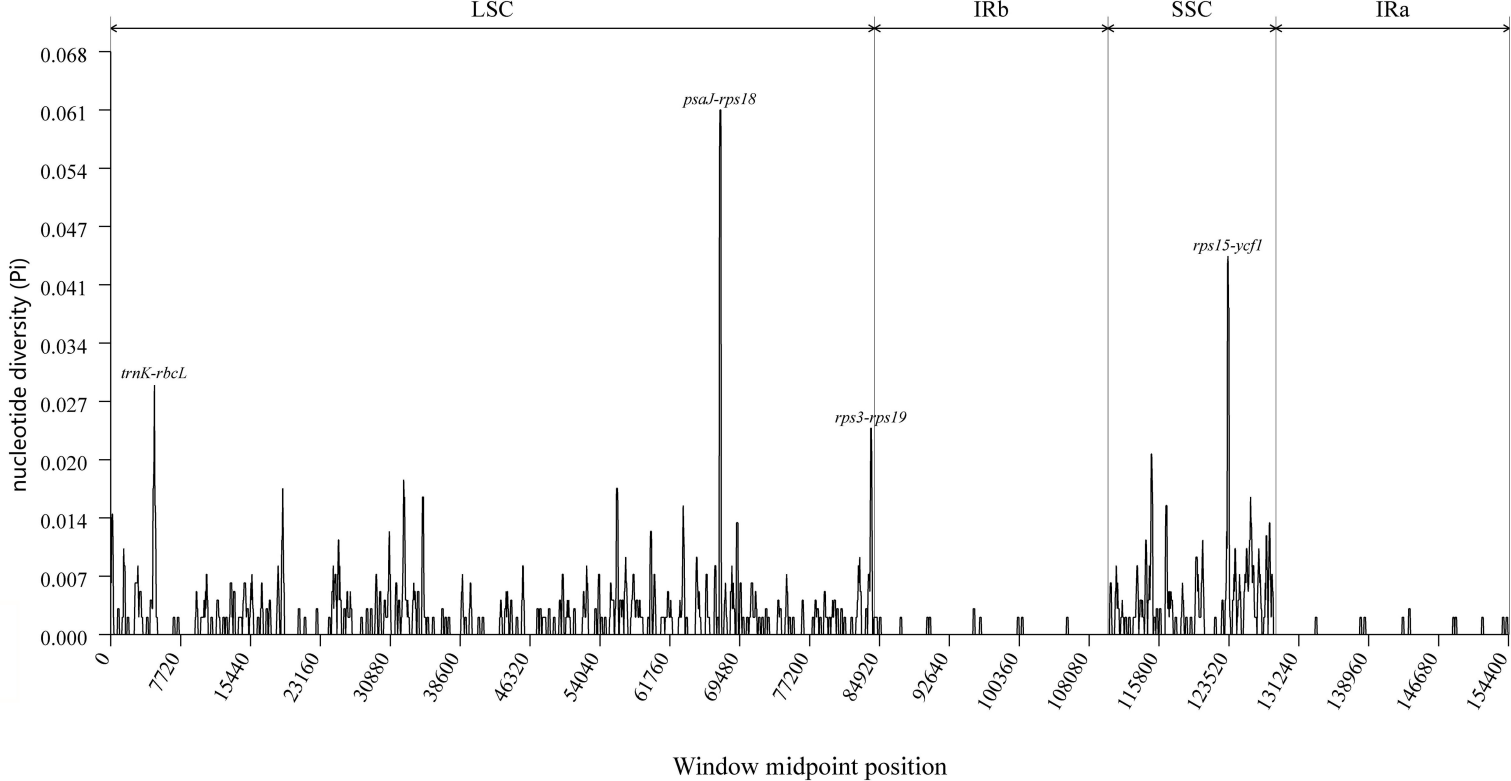
Nucleotide diversity across the complete chloroplast genomes of *F. macrophylla*, *F. prostrata*, *F. stricta*, *Flemingia* sp. and *F. philippinensis*, analyzed using a sliding window approach (window length: 200 bp; step size: 50 bp). The x-axis represents the midpoint position of each window along the genome, while the y-axis indicates the nucleotide diversity (Pi) value for that window.

The DnaSP software was employed to perform sliding window analyses aimed at identifying mutational regions across the complete chloroplast genomes of *F. macrophylla*, *F. prostrata, F. stricta*, *Flemingia* sp. and *F. philippinensi*s. A total of 561 polymorphic sites were identified among the five plastomes, yielding nucleotide diversity (Pi) values ranging from 0 to 0.061, with an average of 0.00167. As illustrated in **Figure 8**, four divergent hotspots exhibiting high Pi values (>0.025) were detected among the five *Flemingia* species: *trnK/rbcL*, *psaJ-rps18*, *rps3-rps19*, and *rps15/ycf1*. All these regions were intergenic spacers, suggesting that intergenic regions are more variable than coding regions. Overall, the LSC and SSC regions exhibited higher divergence compared to the IR regions.

**Figure 9:**
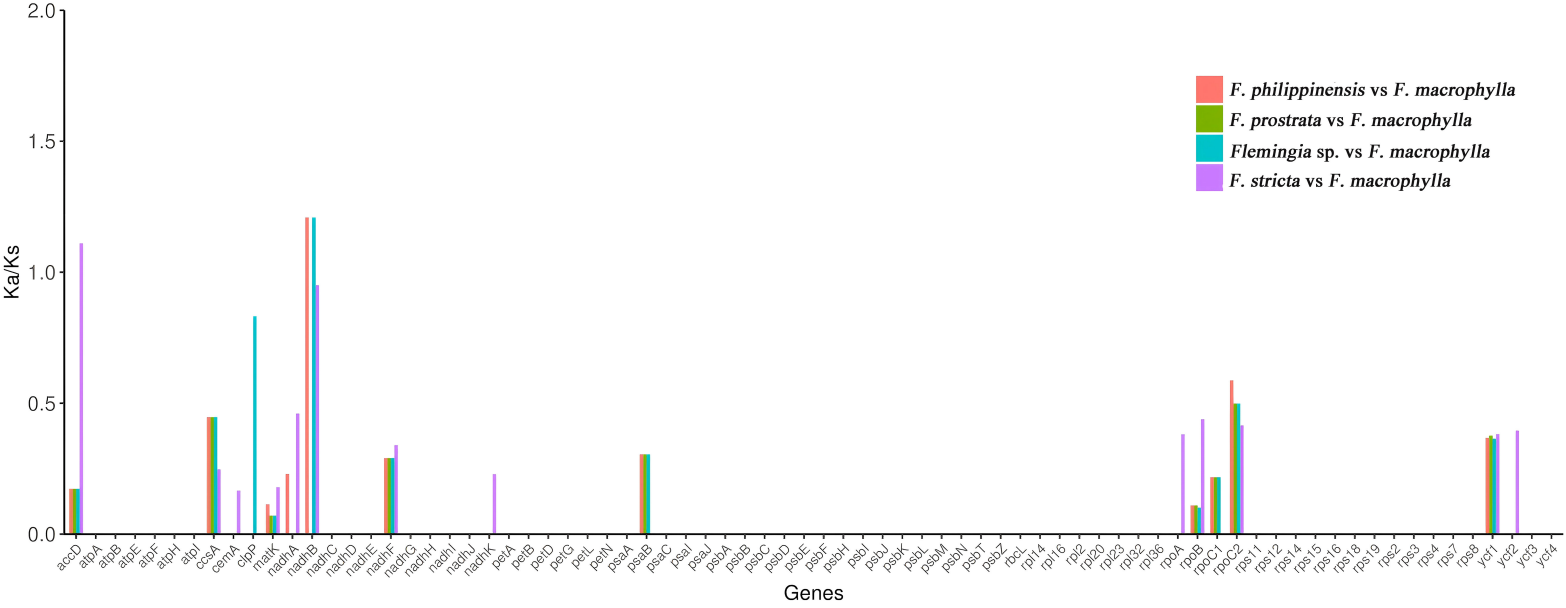
Ka/Ks values of 76 protein-coding genes in the *F. prostrata* vs *F. macrophylla*, *F. stricta* vs *F. macrophylla*, *Flemingia* sp. vs *F. macrophylla*, and *F. philippinensis* vs *F. macrophylla* comparisons.

To identify selection patterns in the protein-coding genes, we compared non-synonymous (Ka) and synonymous (Ks) substitution rates across the five *Flemingia* cp genomes, using *F. macrophylla* as the reference species.The Ka/Ks ratios were calculated for all 76 protein-coding genes. The Ka values for *F. prostrata*, *F. stricta*, *Flemingia* sp., and *F. philippinensis* ranged from 0.00035 to 0.00582, while the Ks values ranged from 0.00169 to 0.05368. The *accD* and *ndhB* genes showed differential selection patterns: *accD* was under positive selection in the *F. stricta* vs *F. macrophylla* comparison (Ka/Ks >1), but under purifying selection in the other three comparisons (Ka/Ks < 1). In contrast, *ndhB* showed no significant difference in the *F. prostrata* vs *F. macrophylla* comparison, was under purifying selection in the *F. philippinensis* vs *F. macrophylla* and *Flemingia* sp. vs *F. macrophylla* comparisons, and also under purifying selection in the *F. stricta* vs *F. macrophylla* comparison. Protein-coding genes inferred to be under purifying selection (Ka/Ks < 1) across all five *Flemingia* chloroplast genomes include *ccsA*, *ycf1*, *nadhF*, *rpoB*, *matK*, and *rpoC2* (**Figure 9**; **Supplementary Table S6**).

### 3.6 SNP and indel in *F. prostrata* and *F. macrophylla*

Comparative analysis of SNPs and indels in the *F. prostrata* cp genome relative to *F. macrophylla* identified 286 SNPs. Among these, 223 were transversional (Tv) and 63 were transitional (Ts), yielding a Ts/Tv ratio of 0.28. The predominant substitution types were G→T (47) and A→C (43), followed by T→A (34), T→G (34), and C→A (31) (**Supplementary Table S7**). Additionally, 104 indels (51 insertions, 53 deletions) were detected. Most indels (68.3%) were 1–5 bp in length, constituting the primary length variation between the two *Flemingia* genomes (**Supplementary Table S8**). These variant sites may serve as candidate molecular markers for species identification and phylogenetics.

### 3.7 Phylogenetic relationships of *Flemingia*

**Figure 10:**
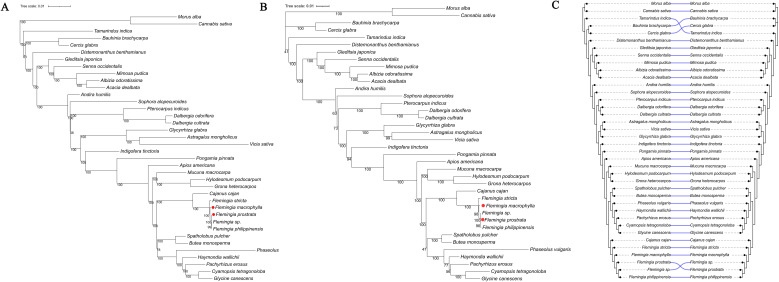
Phylogenetic relationship of the family Fabaceae. **(A)** ML tree based on the CDS of 57 chloroplast genes. **(B)** ML tree based on rbcL + matK + trnL–trnF + ndhF regions. **(C)** The tanglegram compares the two phylogenies, with all branch lengths standardized. Semi−transparent blue curved connectors link each taxon across the two trees; the near−absence of crossing lines indicates a high degree of topological concordance between these datasets.

The two phylogenetic tree indicated that all five *Flemingia* species formed a monophyletic clade, showing a close evolutionary relationship with *Cajanus cajan*, supported by a bootstrap value of 100%. This clade was further grouped with another clade comprising *Glycine canescens*, *Cyamopsis tetragonoloba*, *Pachyrhizus erosus*, *Haymondia wallichii*, *Phaseolus vulgaris*, *Butea monosperma*, and *Spatholobus pulcher* (**Figures 10A, B**).

The tanglegram showed that the vast majority of taxa—including *Morus alba*, *Cannabis sativa*, *Distemonanthus benthamianus*, *Senna occidentalis*, *Glycyrrhiza glabra* and *Phaseolus vulgaris*—were linked by straight, non−crossing semi−transparent blue lines, reflecting identical placements in both the 57−gene CDS tree and the concatenated *rbcL* + *matK* + *trnL*–*trnF*+ *ndhF* tree. Only two small regions displayed minor topological discordance: within the clade comprising *Bauhinia*, *Cercis* and *Tamarindus*, the relative positions of *Tamarindus indica*, *Bauhinia brachycarpa* and *Cercis glabra* were permuted between the two phylogenies; and at the base of the *Flemingia* clade, an unassigned *Flemingia* sp. and *Flemingia prostrata* swapped places, producing a pair of crossing connectors (**Figure 10C**). Overall, although fewer than 10% of terminals were affected and the two datasets exhibited high concordance, the 57−gene dataset showed higher node support, further underscoring the necessity of including more genes in phylogenetic analyses.

### 3.8 Divergence time estimation of *Flemingia*

**Figure 11:**
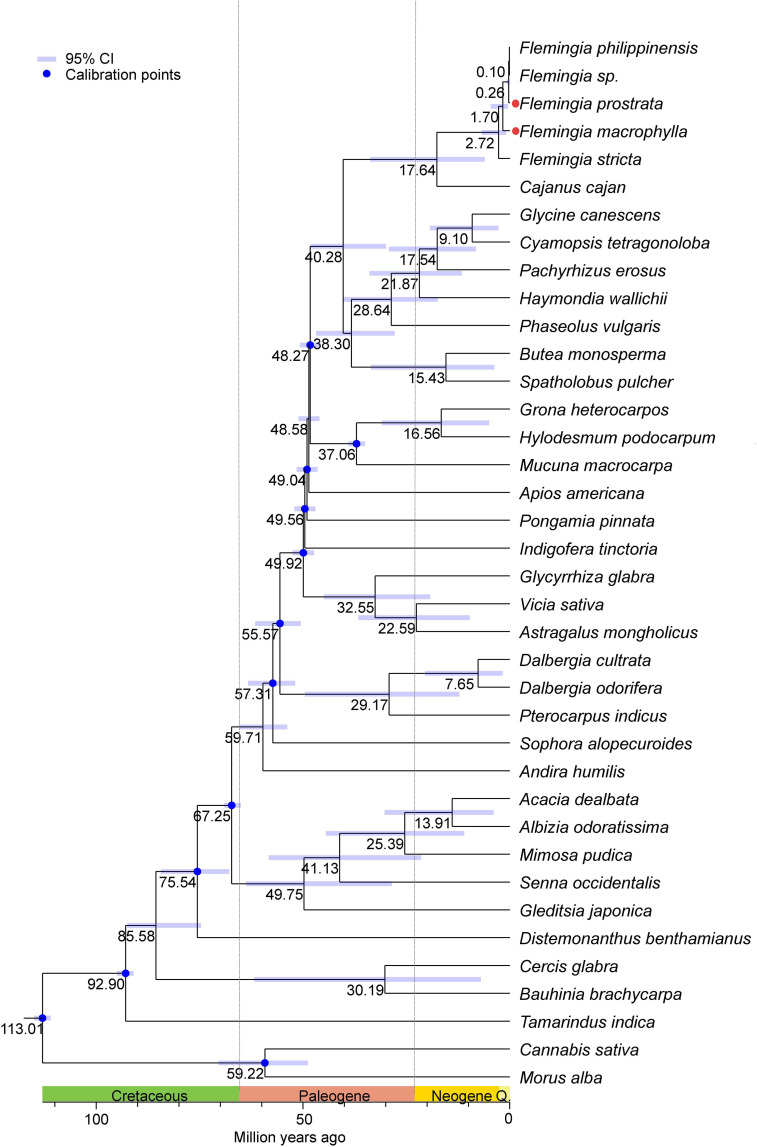
Divergence times estimation based on the 38 complete chloroplast genomes. The numbers near the nodes represent the divergence time (Mya: millions of years ago). Blue indicates the node used to correct the clock. divergence time of the nodes is shown at the nodes with black. The blue bars correspond to 95% HPD of estimated divergence time, with minimum and maximum values, respectively.

Divergence time estimation was conducted using thirty-six chloroplast genomes from Fabaceae species, along with two outgroup genomes from *Cannabis* and *Morus*. The results indicate that the initial divergence of Fabaceae lineages occurred during the Cretaceous period. Within the genus *Flemingia*, divergence times range from 2.72 to 0.10 million years ago (Mya), suggesting that all speciation events took place during the Neogene period. Notably, a rapid divergence occurred within a relatively short evolutionary timeframe. Specifically, *F. macrophylla* and *F. prostrata* diverged approximately 0.26 Mya (**Figure 11**).

### 3.9 SSR molecular identification of *F. macrophylla* and *F. prostrata*

**Figure 12:**
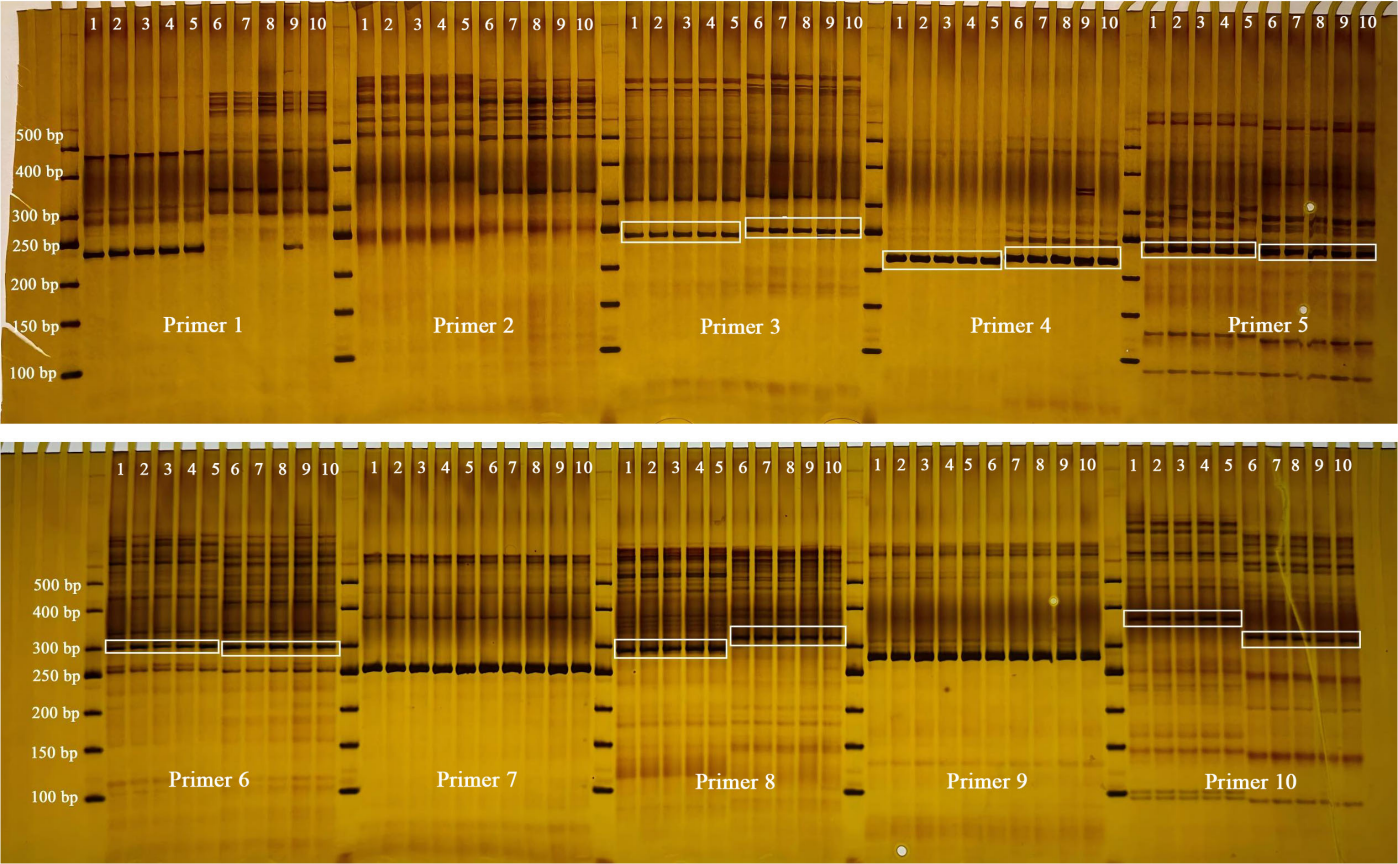
cpSSR electrophoresis results. Lane 1-5: Individual plants of *F. prostrata*; Lane 6-10: Individual plants of *F. macrophylla*.

To effectively distinguish between the two target species (*F. macrophylla* and *F. prostrata*), ten primer pairs were designed and synthesized based on chloroplast SSR (cpSSR) information. Subsequently, PCR amplification and gel electrophoresis were conducted. The results indicated that six primer pairs (Primer 3, Primer 4, Primer 5, Primer 6, Primer 8, and Primer 10) successfully differentiated the two species (**Figure 12**).

## 4 Discussion

In this study, the cp genomes of *F. macrophylla* and *F. prostrata*, with lengths ranging from 152,937 to 153,033 bp (**Table 1**), were sequenced using the Illumina NovaSeq 6000 platform. Both cp genomes showed a typical quadripartite structure, and this was consistent with previous studies on other angiosperms (Liu et al., 2022). Moreover, each *Flemingia* species cp genome encoded 129 genes (8 ribosomal RNAs, 83 coding sequences, 37 transfer RNAs, and 1 pseudogene) (**Table 2**). The genome features, including genome length, gene count, GC content, and SSR patterns, were similar to those of other Fabaceae members (Feng et al., 2022).

Studies on cp genomes have demonstrated the important role of repetitive sequences in events such as duplication, deletion, and rearrangement, and have also investigated phylogeny and genome recombination in Schisandraceae (Li and Zheng, 2018). Here, the repeat sequence analysis showed that a total of 60 and 64 repeat sequences were identified in *F. macrophylla* and *F. prostrata*, respectively, both predominantly ranging from 30–35 bp in length. The differences in repeat sequences may offer valuable insights for the development of molecular markers and the phylogenetic differentiation of the two *Flemingia* species. cpSSRs, as significant molecular markers, exhibit a greater classification distance than nuclear and mitochondrial microsatellites. This feature has been extensively utilized in studies concerning plant population genetics, polymorphism, and evolution (Yue et al., 2023). At present, the application of cpSSR has been reported in *Liriodendron chinense* (Li et al., 2020), *Panicum virgatum* (Zalapa et al., 2011), and *Mimosa scabrella* (Saiki et al., 2017). In this study, 669 SSRs were identified, including mono-, di-, tri-, and tetranucleotide repeats, and these SSRs in cp genomes frequently contained mononucleotide repeats of A/T bases. It was consistent with previously published cp genomes of other plants (Huang et al., 2022). Furthermore, we also analyzed the distribution of SSRs in the cp genomes of two *Femingia* species. Most SSRs were identified in the intergenic regions of the LSC regions, whereas they were predominantly located in the exon of the IR region, suggesting an uneven distribution of SSRs and demonstrating that the non-coding region exhibited greater variability than the coding region in the cp genomes of two *Femingia* species. This aligns with previous reports on the variability of non-coding regions in cp genomes of other angiosperms (Chen et al., 2015).

The expansion and contraction of the IR, LSC, and SSC regions, which are common phenomena for the evolution process, result in variations in the length of cp genomes (Gu et al., 2020). Here, within the five *Flemingia* species, *rps19*, *ycf1*/*ndhF*, and *ycf1* were present at the junction of JLB, JSB, and JSA regions, respectively, while *rpl2* and *trnH* were nearly located on both sides of JLA (**Figure 6**). It was indicated that the boundary of IR, LSC, and SSC regions of five *Flemingia* species was relatively conservative. Meanwhile, we also observed some variations in the boundary between IR and LSC or SSC of the cp genomes of five *Flemingia* species, suggesting differences in their genome lengths.

Codon bias is recognized as an outcome of natural selection, mutation, and genetic drift in diverse organisms (Hong et al., 2020). Two *Flemingia* species showed the same codon, however, variations were observed in the quantity and composition of codons, with distinct preferences among them. Most amino acids exhibited a preference for codons with A/U as the third nucleotide (**Supplementary Table S4**). It was consistent with the research findings of Tang et al. (2022). In addition, our findings indicated significant similarity in the RSCU values between the two *Flemingia* species, suggesting that they may undergo similar environmental pressures.

The cp genomes contain extensive genetic information, offering valuable insights into plant phylogenetic relationships (Jansen et al., 2007). Advances in complete cp genome sequencing have established new platforms and frameworks for systematic studies of medicinal plants. In recent years, publications of cp genomes from multiple Fabaceae subfamilies, including *Caesalpinioideae*, *Papilionoideae*, *Cercidoideae*, *Duparquetioideae*, *Detarioideae*, and *Dialioideae*, have generated critical data for reconstructing the evolution and phylogeny of medicinal Fabaceae species. Our cp genome-based phylogeny groups *F. macrophylla* and *F. prostrata* (both documented medicinal plants in the Chinese Pharmacopoeia) within *Caesalpinioideae*, with *Cajanus cajan* as their closest relative (**Figure 10**). This aligns with prior nuclear gene analyses of 333 Fabaceae genera, which proposed a close relationship between *F. macrophylla* and a clade containing *Dolichos*, *Dunbaria*, *Cajanus*, and *Rhynchosia* (Zhao et al., 2021). Our study demonstrated that the phylogenetic results based on the CDS of 57 chloroplast genes were robust, providing an important reference for molecular phylogenetic studies of Fabaceae at the generic level, and also indicating the necessity of including additional molecular markers in phylogenetic analyses within the genus *Flemingia*. The results obtained here can serve as a valuable reference for molecular phylogeny research in the Fabaceae family, specifically about the contributions of *F. macrophylla* and *F. prostrata* to plant systematics and evolution. These results provide a robust molecular phylogenetic reference for Fabaceae research, particularly regarding the systematic and evolutionary significance of *F. macrophylla* and *F. prostrata*.

Despite clear morphological distinctions between *F. macrophylla* and *F. prostrata*, their dried medicinal materials are challenging to identify in commercial markets. Moreover, their chloroplast genomes remain uncharacterized. Thus, we performed *de novo* assembly of chloroplast genomes via high-throughput sequencing and developed species-specific authentication markers using identified SSR loci. Six cpSSR primer pairs successfully discriminated the two species (**Figure 12**). This study lays the foundation for enriching the molecular data and phylogenetic insights of this genus, as well as for the safe application of medicinal materials.

## 5 Conclusions

In this study, the cp genomes of *F. prostrata* and *F. macrophylla* were sequenced and analyzed. The results revealed that both cp genomes exhibited conserved genome structures, gene information, GC content, codon usage patterns, SSRs, and long repeats. A total of 669 SSRs and 124 scattered repeats were identified across the cp genomes of the two *Flemingia* species, while 286 SNPs and 104 indels were detected between the two cp genomes. Selection pressure analysis indicated that nine genes were under purifying selection. Nucleotide variability analysis revealed that the *psaJ-rps18* displayed the highest level of variation. Phylogenetic analyses supported a close evolutionary relationship between *Flemingia* and *Cajanus*. Divergence time estimation suggested that *F. macrophylla* and *F. prostrata* diverged approximately 0.26 Mya. Six cpSSR primer pairs effectively differentiated the two species. This study provides molecular evidence for distinguishing *F. prostrata* and *F. macrophylla*, contributing to the enrichment of molecular resources and phylogenetic understanding of the genus *Flemingia*, and supporting the accurate identification and safe use of its medicinal materials.

## Data Availability

The datasets presented in this study can be found in online repositories. The names of the repository/repositories and accession number(s) can be found in the article/**Supplementary Material**.
